# Aldosterone Blocks Rat Stem Leydig Cell Development *In Vitro*

**DOI:** 10.3389/fendo.2018.00004

**Published:** 2018-01-24

**Authors:** Jingwei Zhang, Bisheng Huang, Guanghui Hu, Xiangcheng Zhan, Tiancheng Xie, Saiyang Li, Xiaolu Zhang, Huitao Li, Ren-Shan Ge, Yunfei Xu

**Affiliations:** ^1^Department of Urology, Shanghai Tenth People’s Hospital, Tongji University, Shanghai, China; ^2^Department of Urology, Shanghai Tenth People’s Hospital, Nanjing Medical University, Nanjing, China; ^3^Center of Scientific Research, The Second Affiliated Hospital and Yuying Children’s Hospital of Wenzhou Medical University, Wenzhou, China

**Keywords:** mineralocorticoid, aldosterone, stem Leydig cells, proliferation, differentiation, testosterone

## Abstract

Aldosterone (ALDO) is a primary endogenous mineralocorticoid, appearing as the main hormone controlling sodium and water homeostasis. Its emerging role in the development of many organs has gained interest over the past few years. In the testis, Leydig cells contain mineralocorticoid receptors and ALDO stimulates androgen synthesis *via* the mineralocorticoid receptors in rat adult Leydig cells. Although ALDO pharmacologically promoted the Leydig cell function, its role in Leydig cell development was unclear. In the present study, we investigated effects of ALDO on rat stem Leydig cell (SLC) proliferation and differentiation. Using an *in vitro* culture system of the seminiferous tubules from Leydig cell-depleted testis and EdU (a modified thymidine analog) incorporation into the SLC for flurorescent labeling to judge its DNA synthesis and measurement of medium testosterone production, steroidogenesis-related gene and protein expression, we found that: (1) ALDO suppressed EdU incorporation into SLCs at 100 nM *via* mineralocorticoid receptor-mediated mechanism and (2) ALDO reduced Leydig cell number. In conclusion, ALDO pharmacologically blocked rat SLC development.

## Introduction

Aldosterone (ALDO) is a cholesterol-derived steroid hormone and is a primary endogenous mineralocorticoid, controlling sodium and water homeostasis. ALDO binds to the mineralocorticoid receptor (NR3C2), a nuclear transcription factor, which undergoes hormone-dependent nuclear translocation and binds to specific hormone responsive elements, leading to the transactivation of various target genes (1). Besides its main regulation of water and sodium balance, its emerging roles in kidney, brain, heart and lung development has gained interest over the past few years.

In an early study, it was found that ALDO blocked the prepubertal development of testis and the differentiation of Leydig cells without causing atrophic changes in the interstitial elements (2), although the exact mechanism was unclear. Clinically, gonadal dysfunction, such as low sperm count and reduced motility, impotence, decreased libido, and infertility, were occasionally observed in patients undergoing long-term hemodialysis, whose serum testosterone was very low (3). Based on clinical findings, Adachi and Nakada found that five of six nephrectomized rats with renal failure had low serum testosterone levels and that ALDO level was remarkably high in the chronic renal failure rats after surgical ablation of more than about 80% of renal tissues, suggesting that enhanced renin–angiotensin–ALDO system contributes to gonadal impairment (4).

Interestingly, it was found that rat Leydig cells expressed NR3C2 and that ALDO stimulated testosterone production in adult Leydig cells *via* NR3C2 (5). A previous study also found a decreased plasma testosterone level after the ALDO receptor inhibitor spironolactone administration in men (6). This difference between prepubertal period and adulthood suggests that ALDO might have effects on stem Leydig cell (SLC) development.

Conceptually, the pubertal development *in vivo* of rat Leydig cells is divided into four stages: stem, progenitor, immature, and adult Leydig cells (7). This developmental process of Leydig cells can be mimicked in an established *in vitro* culture system, in which SLCs on the surface of the seminiferous tubules following ethane-dimethane-sulfonate (EDS)-induced Leydig cell deleption *in vivo* can be induced into the Leydig cell lineage after 14–21 days of culture (8, 9). The SLCs locate on the surface of the seminiferous tubules and they can either enter the proliferation or commit into the Leydig cell lineage in the presence of different growth factors (8, 9). SLCs can commit into the Leydig cell lineage, by expressing LH receptor (LHCGR, encoded by *Lhcgr*), high-density lipoprotein receptor (SCARB1, encoded by *Scarb1*), steroidogenic acute regulatory protein (StAR, encoded by *Star*), and androgen-biosynthetic enzymes, including cytochrome P450 cholesterol side-chain cleavage enzyme (CYP11A1, encoded by *Cyp11a1*), 3β-hydroxysteroid dehydrogenase 1 (HSD3B1, encoded by *Hsd3b1*), cytochrome 17α-hydroxylase/20-lyase (CYP17A1, encoded by *Cyp17a1*), 17β-hydroxysteroid dehydrogenase 3 (HSD17B3, encoded by *Hsd17b3*), 11β-hydroxysteroid dehydrogenase 1 (HSD11B1, encoded by *Hsd11b1*), and steroid 5α-reductase 1 (SRD5A1, encoded by *Srd5a1*) (7). In the present study, we examined the effects of ALDO on rat SLC proliferation and differentiation *in vitro*.

## Materials and Methods

### Chemicals

Aldosterone and RU28318 were purchased from Steraloids (Newport, RI, USA). Platelet-derived growth factor BB (PDGF-BB) was purchased from Sigma (St. Louis, MO, USA). DMEM/F-12 and Medium 199 culture media were purchased from Invitrogen (Carlsbad, CA, USA). LH was a gift of NIDDK (US). EDS was purchased from Pterosaur Biotech (Hangzhou, China).

### Animals

Ninety-day-old (adult) male Sprague-Dawley rats were purchased from Shanghai Laboratory Animal Co. Ltd. (Shanghai, China) and were raised in a 12 h dark/light cycle temperatures at 23 ± 2°C, and relative humidity of 45–55%. Eighteen rats were adjusted for a week before they were subjected to the intraperitoneal injection (i.p.) of EDS (75 mg/kg). This dose was used to completely deplete Leydig cells according to the previous study (10). Animal procedures were approved by the Institutional Animal Care and Use Committee of Tongji University and were performed in accordance with the Guide for the Care and Use of Laboratory Animals by the National Research Council.

### Seminiferous Tubule Isolation and Culture

Seven days after single i.p. of EDS when all Leydig cells were eliminated (11), seminiferous tubules were mechanically separated from the interstitium as previously described (11). The seminiferous tubules were distributed randomly into 12-well plates, with each well containing tubule fragments of equal total length (about 3 cm). Tubules were cultured at 34°C and 5% CO_2_ in a basal medium (BM), which has 1:1 mixture of DMEM/F-12 and Medium 199 (pH 7.2), sodium bicarbonate (2.2 mg/mL), bovine serum albumin (1 mg/mL), and penicillin (100 U/mL)/streptomycin (100 µg/mL). ALDO (0–100 nM), and/or RU28318 (1 µM) were added to BM to investigate the effects on the proliferation of SLCs. RU28318 is a specific antagonist of NR3C2 (5).

The Leydig cell differentiation-inducing medium (LIM) was constructed by including 5 mM ITS (insulin, transferrin, and selenium), 5 ng/ml LH, and 5 mM lithium chloride as described (11). The previous study showed that seminiferous tubules in LIM were cultured for 14–21 days and SLCs on the surface of seminiferous tubules were fully differentiated into the Leydig cell lineage (8, 11, 12). In this LIM, addition of ALDO (0–100 nM) and/or RU28318 (1 µM) (a NR3C2 antagonist) was performed to investigate the effects of ALDO on SLC differentiation and its mechanism. Concentrations of ALDO and RU28318 were selected according to the previous studies (5, 13). ALDO or RU21318 was dissolved in DMSO and an aliquot of ALDO or RU21318 was added to medium with the final concentration of DMSO as 0.1%. Medium containing 0.1% DMSO served as the control.

Duplicated wells were used at each time point, and each experiment was replicated at least three times.

### Medium Testosterone Analysis

Medium concentrations of testosterone were measured by IMMULITE^®^ 2000 Total Testosterone Immunoassay Kit from Siemens (Gwynedd, UK). The duplicate assay for measurement of testosterone concentration in each medium was performed. The lower detection limit of testosterone was 0.2 ng/mL. Blank medium and blank medium containing 100 nM ALDO or 1 µM RU21318, or 100 nM ALDO + 1 µM RU21318 were measured to check whether they influence the measurement of testosterone levels. No influence was found. The intra-assay and inter-assay CVs were 5.75 and 7.53%, respectively.

### 3β-Hydroxysteroid Dehydrogenase Enzymatic Staining to Label Leydig Cells

Seminiferous tubules were cultured in BM or LIM for 14 days, and tubules then were air dried. Leydig cells on the surface of the tubules were evaluated by histochemical staining for HSD3B1 activity as described previously (14). Briefly, an aliquot of staining solution (25 µL), containing 0.4 mM etiocholanolone as the steroid substrate and 2 mM NAD^+^ as the cofactor and nitro blue tetrazolium chloride as the staining substrate, was added on seminiferous tubules. The samples were incubated at room temperature for 30 min. After staining, seminiferous tubules were washed using PBS and fixed with 4% paraformaldehyde at room temperature for 30 min. The tubules were visualized in a light microscope.

### EdU Incorporation into SLCs

Stem Leydig cell adheres to the surface and its DNA synthesis was measured by the Click-iT^®^ EdU (EDU) Alexa Fluor^®^ 488 Imaging Kit (Life Technologies, OR, USA). A previous study demonstrated that PDGF-BB (10 ng/mL) robustly stimulated DNA synthesis in SLCs (15). Therefore, PDGF-BB (10 ng/mL) was used to increase DNA synthesis in SLCs. The isolated seminiferous tubules were cultured in BM with ALDO and/or RU28318 for the additional 5 days without (control) or with 10 ng/ml PDGF-BB. Then, EdU was supplied to the culture well and the samples were incubated for 24 h. Tubules were washed, fixed in 4% paraformaldehyde, and incubated with reaction solution. EdU incorporation in SLCs was visualized under a fluorescence microscope (Olympus, Tokyo, Japan) and images were captured. EdU-positive cells were counted and calculated by the flat surface area of the seminiferous tubules using the Image Pro Plus 6.0 software (Media Cybernetics, Inc., MD, USA). A total of 20 seminiferous tubule images were counted. Some seminiferous tubules were embedded in 2% argose gel, fixed in 4% paraformaldehyde, and cross sections (10 µm) were cut. Sections were incubated with mouse monoclonal antibody against α-smooth muscle actin (SMA, Sigma, A2547, 1:200) and the conjugated secondary antibody (Donkey anti-Mouse IgG Secondary Antibody, Alexa Fluor^®^ 594, Invitrogen, A-21203, 1:500) was used to visualize SMA. The EdU staining procedure was performed as above. The sections were visualized under immunofluorescence microscope.

### Cell Immunofluorescence to Identify Leydig Cells

Seminiferous tubules were treated with or without ALDO and/or RU28318 in LIM for 14 days and were embedded in 2% agarose gel, and cross sections (10 µm) were cut as previously described (11). In brief, the sections were later fixed in 4% paraformaldehyde. Sections were washed and then incubated with mouse monoclonal anti-SMA antibody (1:200) and rabbit monoclonal antibody against HSD11B1 (Pterosaur Biotech, PB 10021, 1:500) for 60 min followed by incubation with conjugated secondary antibody (Alexa Fluor^®^ 594 for SMA) and (donkey anti-rabbit Dylight 488, Bioworld, BS10018, 1:100 for HSD11B1) for 30 min. HSD11B1 was used to identify Leydig cells at the advanced stage because it begins expressed in rat Leydig cells starting on postnatal day 28 (16), when immature Leydig cells are emerged (17). SMA was used for labeling peritubular myoid cells (11). The sections were counterstained with DAPI (Sigma, D8417, 2 µg/mL). HSD11B1-positive Leydig cells per tubule cross section were counted.

### Staining SLCs Using CD90 Antibody

A previous study showed that CD90^+^ cells on the surface of the seminiferous tubules were SLCs (11). Seminiferous tubules were treated with or without 1–100 nM ALDO in BM for 5 days. The seminiferous tubules were washed using PBS containing 1% BSA. Then, SLCs were stained using PE-conjugated CD90 antibody (Thermo Fisher Scientific, 12-0900-83, 1:100) in the dark place at 4°C for 45 min. The tubules were mounted on a slide for visualization under a fluorescence microscope (Olympus, Japan) and images were captured. CD90-positive cells were counted and calculated by counting SLC number per mm^2^ flat surface area using the ImageProPlus 7.0 software (Media Cybernetics, USA).

### Real-time Quantitative Polymerase Chain Reaction (qPCR)

Seminiferous tubules were treated with or without ALDO and/or RU28318 in LIM for 14 days. Then tubules were washed and submerged to 0.5 mL Trizol^®^ Reagent (Life Technologies, CA, USA), and total RNAs were extracted according to the manufacturer’s instruction as described (11). The Leydig cell genes and their primers were used as described previously (18–20). Primers used for qPCR had at least one span intron. These genes include *Lhcgr, Scarb1, Star*, C*yp11a1, Hsd3b1, Cyp17a1, Hsd17b3, Srd5a1*, and *Hsd11b1*. The relative mRNA levels of targeted genes were normalized to *Rps16* (internal control gene). The RNA was reversely transcribed into cDNA using Reverse Transcription System (Promega, WI, USA) according to the manufacturer’s instruction. The qPCR was carried out in a 25-µl reaction volume with SYBR Green detection system (Bio-Rad Laboratories, Inc., CA, USA). Light Cycler^®^480 SYBR Green I Master was purchased from Roche Diagnostics (IN, USA). Reactions were run on a Bio-Rad qPCR system (Bio-Rad Laboratories, Inc., CA, USA) for up to 40 cycles and the melting curves were routinely checked afterward. The Ct value was recorded and the standard curve method was used to calculate the gene expression levels as previously described (21).

### Western Blotting

Seminiferous tubules were treated with or without ALDO and/or RU28318 in LIM for 14 days. Tubules were washed with PBS and submerged to the Radioimmunoprecipitation Assay Buffer (Beyotime Biotechnology, Shanghai, China) and homogenized to obtain total proteins. The protein concentrations of samples were measured with an Enhanced BCA Protein Assay Kit (Beyotime Biotechnology, Shanghai, China). An aliquot (50 µg of proteins) of sample was added to gel well and electrophoresed on 10% polyacrylamide gels containing sodium dodecyl sulfate and proteins were transferred. Then, the membranes were incubated overnight at 4°C with primary antibodies against the following antigens: SCARB1 (MultiSciences, 70-ab1967-050, 1:1,000), LHCGR (MultiSciences, 70-ab7496-050, 1:1,000), and β-actin (ACTB, Beyotime, AA128, 1:1,000). ACTB (house-keeping protein) served as the internal control. The membranes were then washed and incubated with HRP-conjugated anti-rabbit IgG secondary antibody (MultiSciences, 70-GAR0072, 1:2,000) or HRP-conjugated anti-mouse IgG secondary antibody (MultiSciences, 70-GAM0072, 1:2,000) for 2 h at room temperature and washed. The immunoreactive bands were visualized by chemiluminescence using Western Bright^®^ ECL (Advansta, CA, USA). The intensity of band was analyzed with Image J software 1.51j8 (NIH, USA). The intensity was adjusted and calculated relative to the control (set as 100%).

### Statistical Analysis

Values are expressed as mean ± SD, and data were analyzed by the GraphPad Prism 6 (GraphPad Software Inc., CA, USA). After the normal distribution is confirmed, multiple groups were performed by one-way ANOVA followed by *ad hoc* Tukey’s comparison of all columns compared with the control column. Mean value comparisons between two groups were performed by t-test. Differences were considered significant at *P* < 0.05.

## Results

### ALDO Inhibits SLC Proliferation

**Figure 1:**
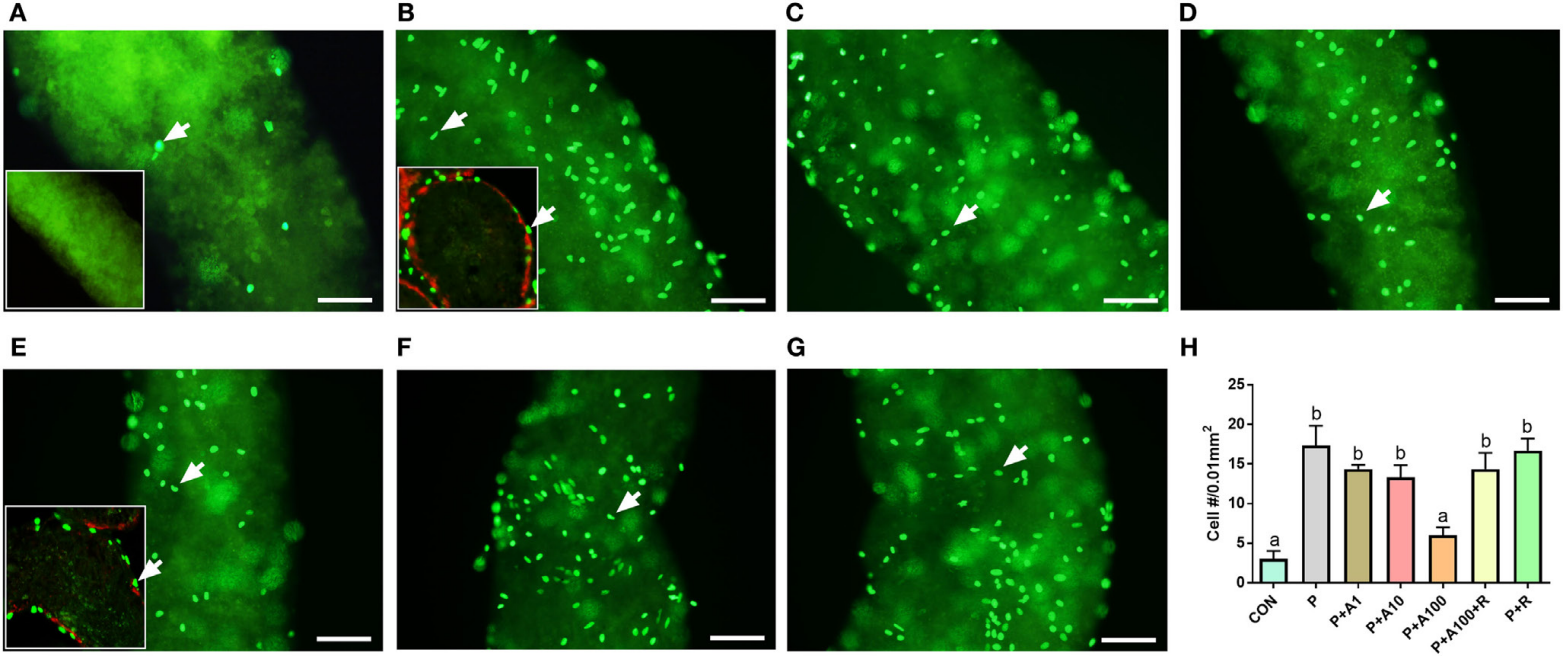
EdU incorporation into stem Leydig cells (SLCs) after aldosterone (ALDO) treatment for 5 days. **(A–G)** EdU-labeled SLCs (green color in nuclei, arrow) in basal medium **(A)**, with Platelet-derived growth factor BB (PDGF-BB) 10 ng/mL **(B)**, with PDGF-BB 10 ng/mL + ALDO 1 nM **(C)**, PDGF-BB 10 ng/mL + ALDO 10 nM **(D)**, PDGF-BB 10 ng/mL + ALDO 100 nM **(E)**, PDGF-BB 10 ng/mL + ALDO 100 nM + RU21318 1 µM **(F)**, and PDGF-BB 10 ng/mL + RU21318 1 µM **(G)**. **(H)** Quantitative data of EdU-incorporated cell number; bar = 40 µm. **(A)** Inset image presents negative control. **(B,E)** Inset images present the EdU incorporation in cross section; SMA (red color) represents peritubular myoid cells and green color represents EdU-incorporated cells (white arrow). P = PDGF-BB 10 ng/mL, A1–100 = ALDO 1–100 nM, R = RU21318 1 µM. Mean ± SD, *n* = 3. Identical letters showed no significant difference between two groups at *P* < 0.05.

To investigate the potential effects of ALDO on the proliferation of SLCs, we adopted an *in vitro* seminiferous tubule culture system. A previous study demonstrated that PDGF-BB (10 ng/ml) significantly increased EdU incorporation into the nuclei of SLCs (11). Isolated seminiferous tubules were cultured for 5 days with PDGF-BB (10 ng/mL) in the presence of 0–100 nM ALDO and/or a NR3C2 antagonist RU28318, then, EdU incorporation into SLCs on the surface of seminiferous tubules was investigated. As shown in Figure 1A, in BM, there are a few EdU-incorporated SLCs present on the surface of the seminiferous tubules. PDGF-BB (10 ng/ml) remarkably increased EdU-incorporated number of SLCs (Figure 1B), which were located on the surface of the seminiferous tubule (outside SMA-positive myoid cells, Figure 1B inset). When PDGF-BB plus 1–100 nM ALDO was used, ALDO concentration-dependently decreased PDGF-BB-stimulated EdU-incorporated SLCs (Figures 1C–E), with 100 nM ALDO being significant difference compared to the control (Figure 1E). RU28318 (the NR3C2 antagonist) alone did not affect PDGF-BB-mediated EdU incorporation (Figure 1G), while it reversed the effect of 100 nM ALDO (Figure 1F). This suggests that ALDO suppresses SLC proliferation *via* binding to NR3C2. Quantitative data of EdU incorporated cell number was summarized (Figure 1H).

**Figure 2:**
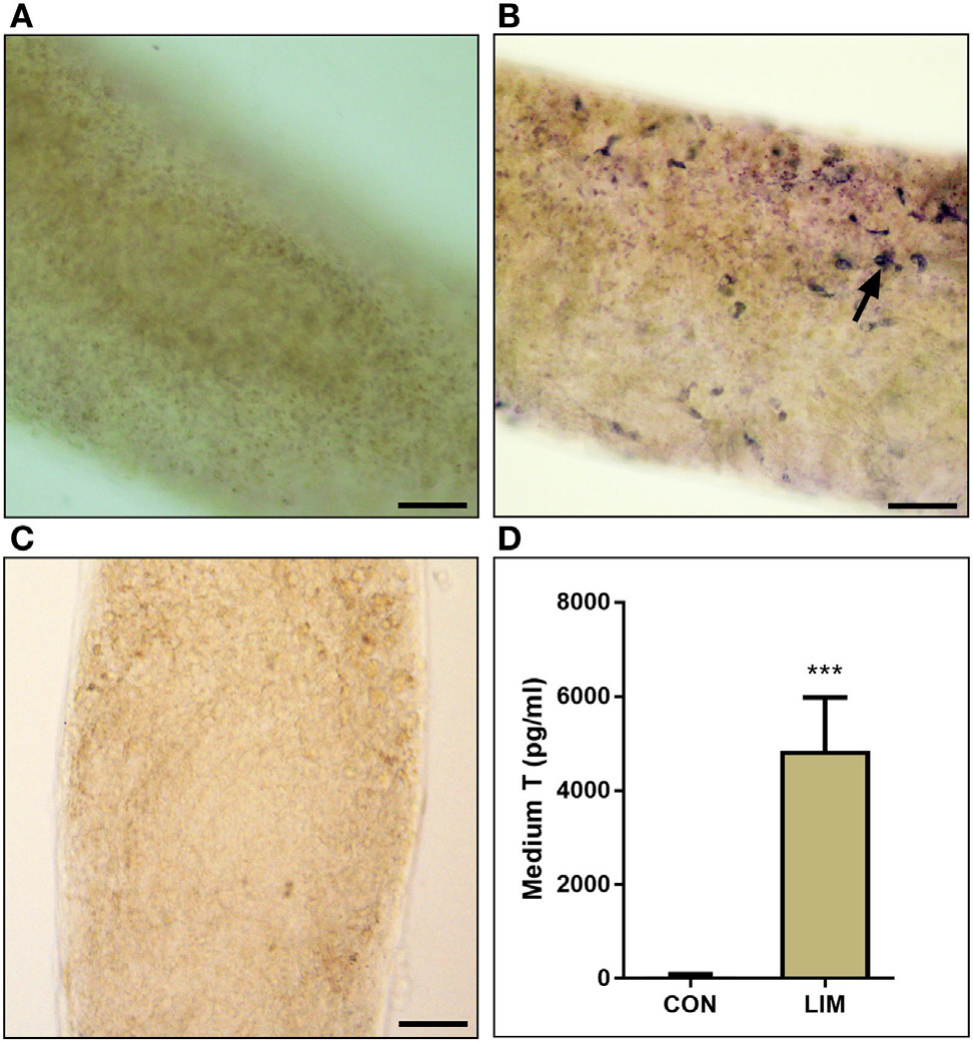
3β-Hydroxysteroid dehydrogenase enzymatic staining to label Leydig cells and testosterone level. **(A)** Seminiferous tubule in basal medium (BM) for 14 days. **(B)** Seminiferous tubule in LIM for 14 days. Black arrow points to Leydig cells stained by 3β-hydroxysteroid dehydrogenase. **(C)** Negative control. Bar = 50 µm. **(D)** Medium T levels in BM (CON) and LIM for 14 days. Mean ± SD, *n* = 6; ***P < 0.001 between control and LIM.

We cultured the seminiferous tubules in BM (control) or LIM for 14 days. No HSD3B1-positive Leydig cells were formed in the control (Figure 2A), while many HSD3B1-positive Leydig cells were formed on the surface of the seminiferous tubule after culture with LIM (Figure 2B). Apparently, significantly higher level of testosterone was produced by the Leydig cells after culture with LIM (Figure 2D). This confirms the previous observation that LIM is an inducer for SLC differentiation.

**Figure 3:**
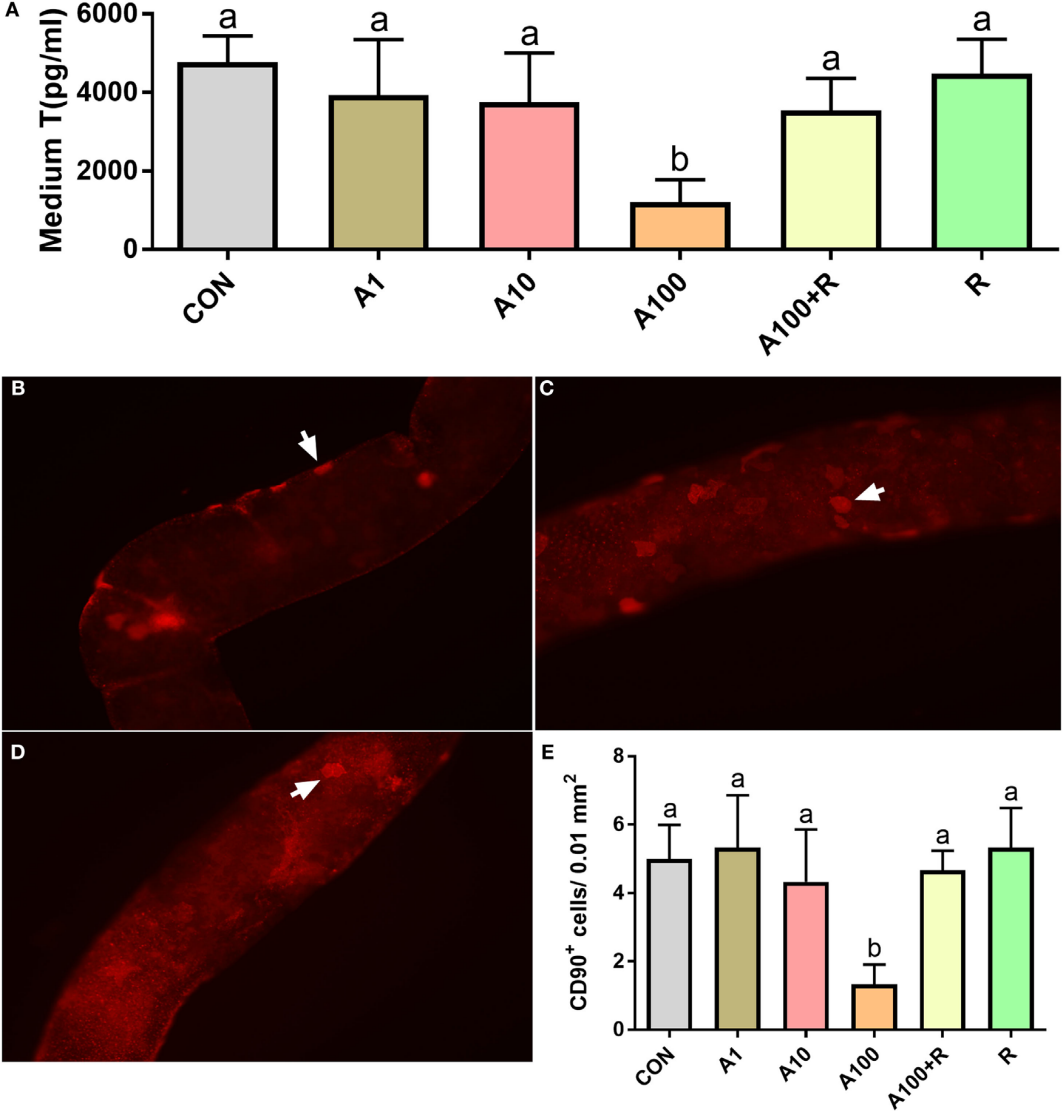
Effects of aldosterone (ALDO) on stem Leydig cell (SLC) proliferation and CD90-positive SLC number. Seminiferous tubules were cultured in basal medium (BM) with various concentrations of ALDO and/or RU28318 for 7 days and then were switched into LIM for additional 14 days, medium testosterone (T) levels were measured (mean ± SD, *n* = 4–8). **(A)** ALDO (0–100 nM) and/or RU21318, A1–100 = ALDO 1–100 nM, R = RU21318 1 µM. Images of CD90-positive cells at ALDO 0 nM **(B)**, at ALDO 10 nM **(C)**, and ALDO 100 nM **(D)** in BM for 5 days; white arrow points to CD90-positive SLC. **(E)** Quantitative data of CD90-positive cells after ALDO and RU28318 treatment; mean ± SD, *n* = 3. Identical letters showed no significant difference between two groups at *P* < 0.05.

To further examine whether ALDO can affect the pool of SLCs, the seminiferous tubules were cultured in BM with various concentrations of ALDO (0–100 nM) and/or RU28318 for 7 days and then the seminiferous tubules were switched into LIM for additional 14 days to induce these SLCs into the Leydig cells. Testosterone levels in the medium were significantly decreased at 100 nM ALDO (Figure 3A), and its effect was reversed by RU28318, indicating the effect was acted *via* binding to NR3C2 (Figure 3A). A previous study demonstrated that CD90-positive cells were SLCs (11). After 5 days of treatment with 0–100 nM ALDO in BM, we found that ALDO (100 nM) significantly lowered CD90-positive SLC number and the effect was reversed by RU28318 (Figures 3B–E).

### ALDO Affects SLC Differentiation

**Figure 4:**
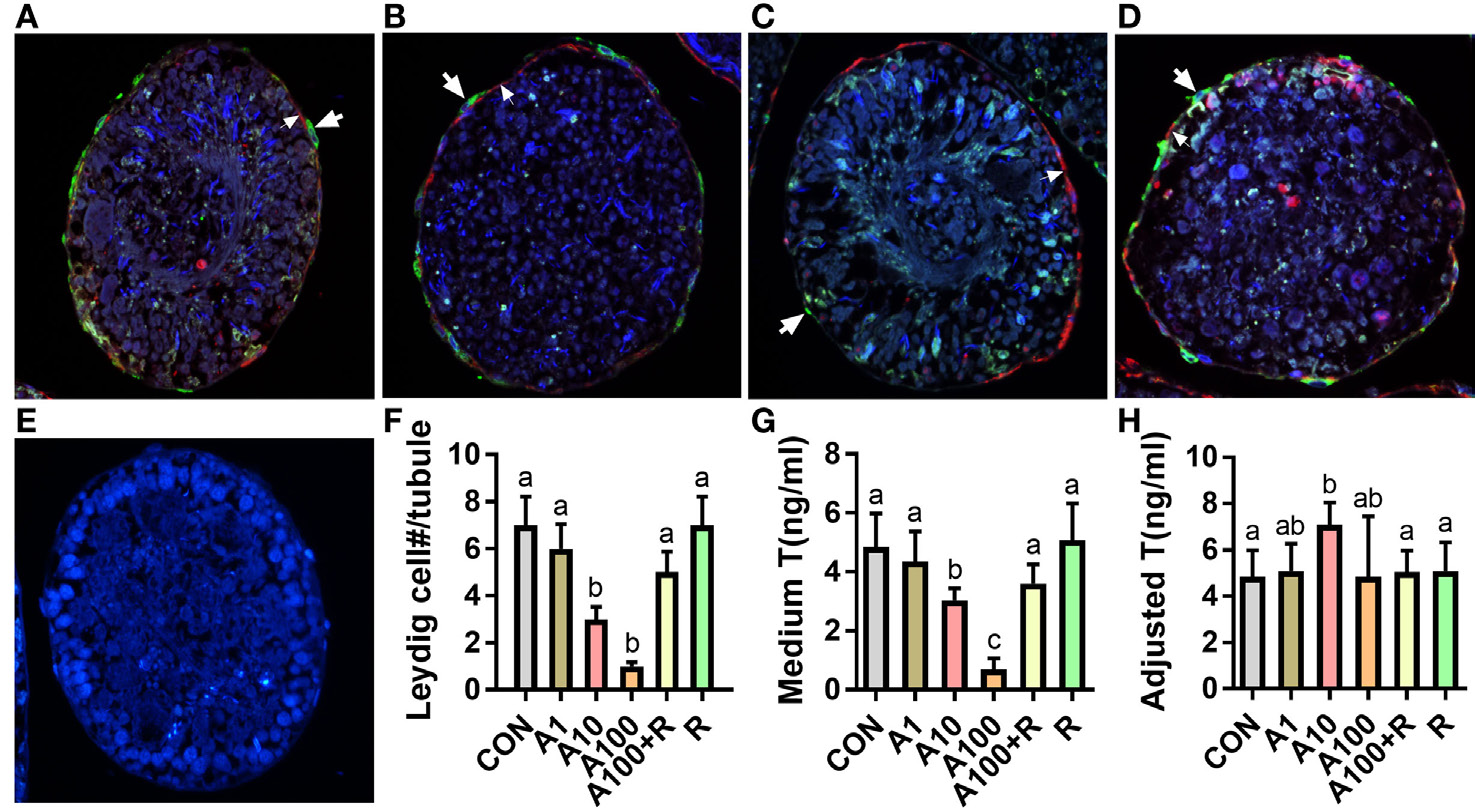
Aldosterone reduces the formation of Leydig cells. **(A–G)** Immunofluorescent staining of tubule cross section after aldosterone (ALDO) and/or RU21318 treatment in LIM for 14 days. Cross sections with HSD11B1 staining (green color, thick arrow) showed the formation of Leydig cells. α-Smooth muscle actin staining (red color, thin arrow) showed the myoid cells, which circled the seminiferous tubules. HSD11B1-positive cells were outside the α-smooth muscle actin-positive cells, indicating that they were differentiated from the stem Leydig cells (SLCs) on the surface of the tubule. **(A)** ALDO 0 nM; **(B)** ALDO 10 nM; **(C)** ALDO 100 nM; **(D)** ALDO 100 nM + RU21318 1 µM; **(E)** negative control; and **(F)** quantitative data of Leydig cell number per cross section; mean ± SD, n = 3. **(G)** Quantitative data of testosterone level after ALDO treatment, mean ± SD, *n* = 6. **(H)** Quantitative data of adjusted testosterone level after ALDO treatment by Leydig cell number, mean ± SD, *n* = 6. Identical letters showed no significant difference between two groups at *P* < 0.05.

The seminiferous tubules were continuously cultured in LIM for 14 days to induce SLCs into adult Leydig cells, thus producing testosterone as in Figure 2D. When various concentrations of ALDO were added in LIM and the seminiferous tubules were cultured for 14 days. ALDO decreased the HSD11B1-positive Leydig cell numbers at ≥10 nM and RU28318 reversed the ALDO-mediated effects (Figures 4A–F). ALDO decreased medium testosterone levels at ≥10 nM and RU28318 (1 µM) alone did not affect the SLC differentiation (Figure 4G). After adjustment by the Leydig cell numbered, it was found that 10 nM ALDO increased medium testosterone levels (Figure 4H). This indicates that ALDO stimulates Leydig cell differentiation of the existing stem cells.

### ALDO Alters Leydig Cell-Specific Genes and Protein Expression

**Figure 5:**
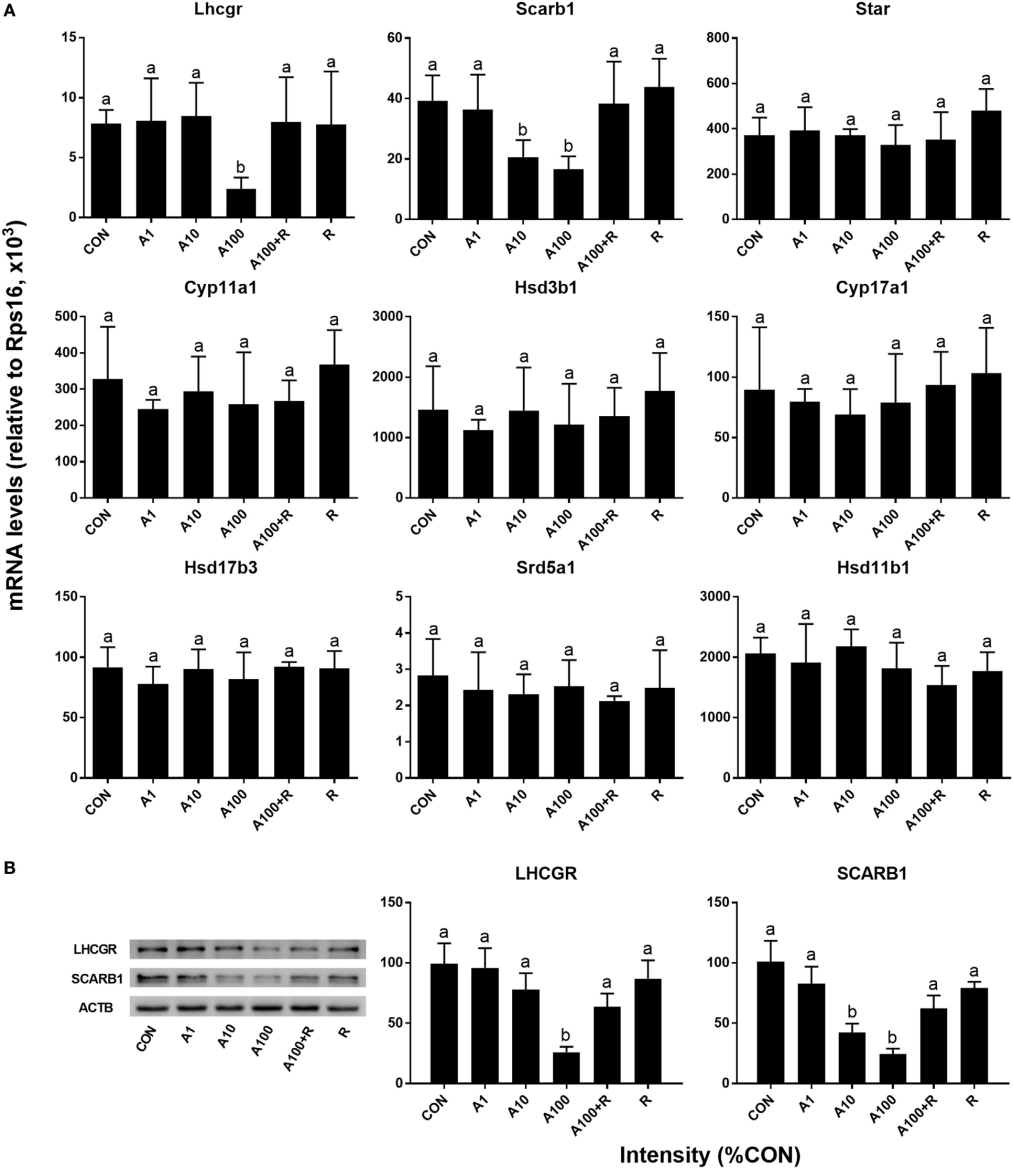
Aldosterone (ALDO) reduces Leydig cell-specific gene and its protein expression levels. Seminiferous tubules were cultured in LIM in the presence of ALDO and/or RU21318 for 14 days, then mRNA levels of Leydig cell-specific mRNA levels were measured by quantitative polymerase chain reaction (*n* = 4–6), and SCARB1 and LHCGR levels were measured by Western blot (*n* = 3). A1–100 = ALDO 1–100 nM, R = RU21318 1 µM. **(A)** mRNA; **(B)** protein; mean ± SD. Identical letters showed no significant difference between two groups at *P* < 0.05.

**Figure 6:**
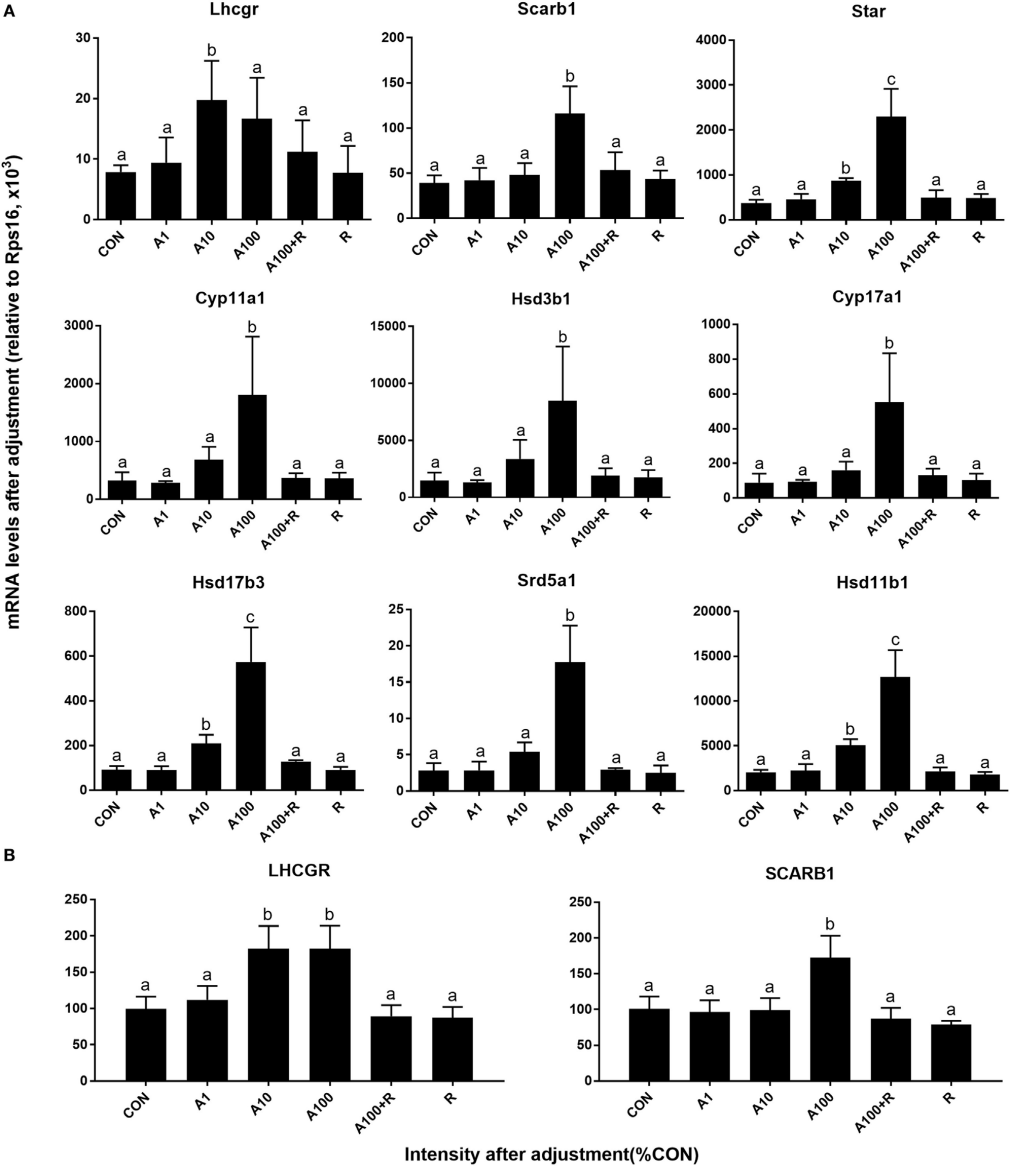
Aldosterone (ALDO) affects Leydig cell-specific gene and its protein expression levels after adjustment of Leydig cell number. A1–100 = ALDO 1–100 nM, R = RU21318 1 µM. **(A)** mRNA after adjustment; **(B)** protein after adjustment; mean ± SD. Identical letters showed no significant difference between two groups at *P* < 0.05.

We examined the effects of ALDO on the expression levels of Leydig cell-specific genes (Figure 5A). Statistically, we found ALDO significantly reduced the *Scarb1* (encoding SCARB1) level at ≥10 nM and *Lhcgr* (encoding LHCGR) level ≥ 100 nM. However, ALDO did not affect other seven gene mRNA levels. However, we further calculated *Lhcgr, Scarb1, Star*, C*yp11a1, Hsd3b1, Cyp17a1, Hsd17b3, Srd5a1*, and *Hsd11b1* levels after adjustment of Leydig cell number (Figure 6) and we found that ALDO actually increased the expression levels of all these genes at 10 nM and higher. In parallel, we also selected two important Leydig-cell specific proteins, SCARB1 and LHCGR, and we found the levels of these two proteins were reduced by ALDO (Figure 5B or Figure S1–S3 adapted to ImageJ Software). However, ALDO increased SCARB1 and LHCGR protein levels per cell after adjustment of Leydig cells (Figure 6). RU28318 reversed ALDO-mediated effects. These results indicate that ALDO inhibits the proliferation of SLCs by lowering Leydig cell number but induces the differentiation of SLCs *via* increasing the Leydig cell specific gene expression.

## Discussion

Mineralocorticoid is pivotal during the perinatal period in many target tissues. In this research, we mainly focused on the developmental specificities of the primary endogenous mineralocorticoid ALDO in rat SLCs. We found that ALDO targeted SLCs to inhibit their proliferation.

In the present study, SLCs in the medium containing ITS, LH, and LI (LIM) could be induced into testosterone-producing Leydig cells *in vitro* (Figure 2), confirming the previous observation (11). In the non-LIM medium, Leydig cells cannot be induced because Leydig cell biomarker CYP11A1 was not expressed (11). Benefiting from this system, we could examine ALDO to regulate SLC differentiation and proliferation without the interference by hypothalamus-pituitary secreted hormones. In this system, we demonstrated that ALDO suppressed the proliferation of SLCs *in vitro via* NR3C2-induced effects, because the NR3C2 antagonist RU28318 could reverse its action.

The present study demonstrated that ALDO suppressed the proliferation of SLCs *in vitro* and ALDO-induced effects. Interestingly, ALDO increased many Leydig cell-specific gene (such as *Lhcgr, Scarb1, Star*, C*yp11a1, Hsd3b1, Cyp17a1, Hsd17b3, Srd5a1*, and *Hsd11b1*) expression level after adjustment of Leydig cell number, suggesting that ALDO stimulates Leydig cell gene expression. Thus, ALDO increased testosterone production in Leydig cells *per se* (Figure 4H). However, ALDO significantly reduced Leydig cell numbers, thus reducing testosterone production in the medium. This conforms to our previous observation that ALDO acutely stimulated testosterone production in adult Leydig cells. Evidently, ALDO acted *via* NR3C2, because the NR3C2 antagonist RU28318 reversed ALDO-mediated alteration of the gene expression of Leydig cell genes such as *Lhcgr, Scarb1, Star*, C*yp11a1, Hsd3b1, Cyp17a1, Hsd17b3, Srd5a1*, and *Hsd11b1* (Figures 5 and 6) and testosterone levels (Figure 4). ALDO affected SLC function in a pharmacological concentration (at ≥10 nM). Indeed, ALDO blocked the development of rat Leydig cells at pharmacological doses (2). Clinically, a decreased plasma testosterone level was found in men with the ALDO receptor inhibitor spironolactone administration (6), and enhanced renin–angiotensin–ALDO system (with increase of ALDO levels) might also contribute to gonadal impairment (4). Other clinical data was lacking and further clinical investigation for ALDO action on Leydig cell development is required.

Interestingly, unlike the effects of ALDO on SLC function as shown in the present study, ALDO stimulated testosterone production in rat adult Leydig cells *via* an MR-mediated mechanism (5). Although ALDO stimulated the Leydig cell specific gene expression and testosterone production *per se*, it significantly reduced testosterone production by decreasing the Leydig cell number. The developmentally different effects that ALDO exerted on Leydig cell development are still unclear. We speculate that the different ALDO-NR3C2 action on Leydig cells at different stages may depend on intracellular NR3C2 binding proteins. Indeed, NR3C2 actions were regulated by chaperone proteins such as heat shock proteins 90 and 70 (hsp90 and hsp70) and immunophylins (22, 23) and posttranslational modifications such as phosphorylation, ubiquitinylation, sumoylation, and acetylation as well as interactions with many coregulators (23).

In conclusion, ALDO inhibited the proliferation of SLCs *via* NR3C2, thus leading to the reduced Leydig cell number and testosterone production, although it stimulated the expression of Leydig cell genes and testosterone production in the single cell.

## Ethics Statement

Animal procedures were approved by the Institutional Animal Care and Use Committee of Tongji University and were performed in accordance with the Guide for the Care and Use of Laboratory Animals by the National Research Council.

## Author Contributions

JZ, BH, GH, XZhan, TX, SL, XZhang, and HL performed experiments; JZ, BH, and GH analyzed data; R-SG and YX designed experiments and wrote the manuscript.

## Conflict of Interest Statement

The authors declare that the research was conducted in the absence of any commercial or financial relationships that could be construed as a potential conflict of interest.
