# Supplementary figures and images for: Aldosterone Blocks Rat Stem Leydig Cell Development In Vitro
Source: Front Endocrinol (Lausanne). 2018 Jan 24;9:4. doi: 10.3389/fendo.2018.00004 (PMC5787991; doi:10.3389/fendo.2018.00004)

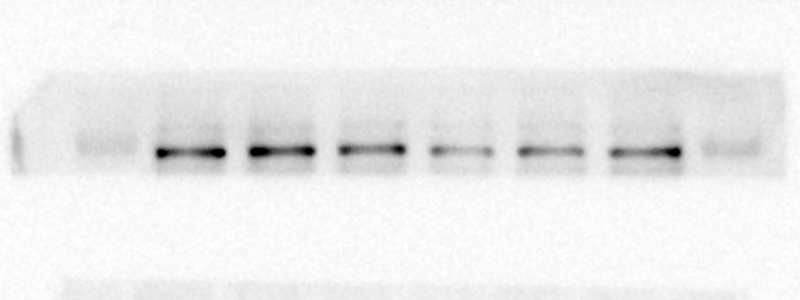

Supplement: Supplementary file 1 [file Image_1.TIF]

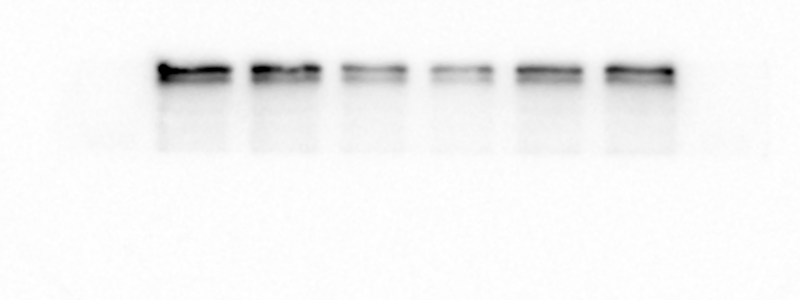

Supplement: Supplementary file 2 [file Image_2.TIF]

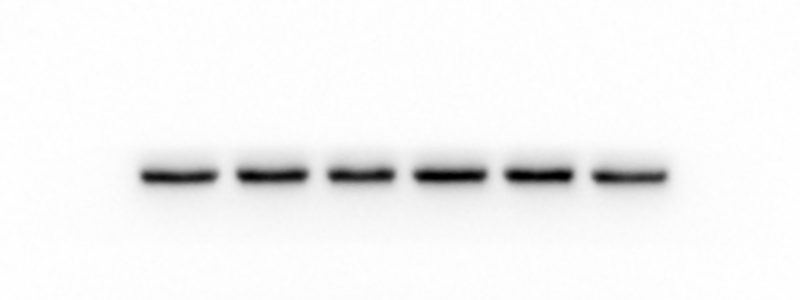

Supplement: Supplementary file 3 [file Image_3.TIF]

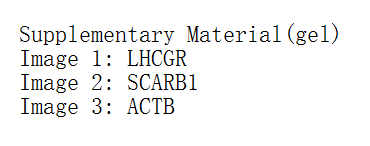

Supplement: Supplementary file 4 [file Image_4.PNG]
